# Meerschaum

**Published:** 1883-01

**Authors:** 


### MEERSCHAUM.

The place most productive of this mineral is known to be near the town of Eski-scheir, in Anatolla, Asia Minor. A recent account by Herr Adler states that the preparation of one hundred boxes of meerschaum there takes twelve to fifteen persons two months, and costs about £120. In Eski-scheir the average cost of the box of mercantile ware has varied since 1873 between about £6 to £10 (last year it was about the former). Refuse ware can be had at about an eighth of the price. There are ten qualities, and each is to be had in four sizes, there being twenty-five to forty pieces of the first size per box, and 450 to 1,500 pieces of the fourth (the box is thirty inches long, eight inches broad and 15½ inches deep. In the last two decades the export of meerschaum has considerably increased; from 3,000 boxes in 1855, it has risen to 11,100 in 1881. In Constantinople the trade is managed by about fifteen firms—Austrian, Bulgarian, Greek, Armenian and Turkish—who bring their wares into the Vienna market. The large importation into Vienna may be said to date from between 1850 and 1860, when the production of pipe-bowls and cigar-tips was greatly increased for export to England, France and North America. In 1860 a considerable export of pipes to San Francisco was first developed, while large quantities of cigar-tips were sent to America and Australia via Hamburg. Since then the conditions of the trade have altered much, chiefly in consequence of the high duties imposed in America. In that country arose, with the aid of emigrant turners from Austria, a home industry, which has successfully competed with the Vienna pipe manufacture (for the products of which America was previously the best customer). With France and Germany, the United States obtains the raw product mainly from Austria.

---

THE famous blue laws of Connecticut, enacted by "the people of the Dominion of New Haven," were so called because printed on blue paper. They prohibited the ceremony of marriage being performed by a parson, on the strange ground that a magistrate might perform it with less scandal to the church. Adultery was punished by death. Wearing clothes trimmed with gold, silver or lace above one shilling a yard involved a tax on the person's estate of $15. "No one to cross a river on the Sabbath but authorized clergymen. No one shall travel, cook, make beds, sweep houses, cut hair or shave on the Sabbath. No one shall kiss his or her children on the Sabbath or fasting days. The Sabbath day shall begin at sunset Saturday."

